# High accuracy breast cancer classification with BIRADS and coclustering

**DOI:** 10.1371/journal.pone.0340772

**Published:** 2026-02-09

**Authors:** Run Zhou, Xujiang Yu, Jianhao Wang

**Affiliations:** School of Mechanical and Electrical Information, Yiwu Industrial & Commercial College, Yiwu, Zhejiang, People’s Republic of China; The University of Texas, MD Anderson Cancer Center, UNITED STATES OF AMERICA

## Abstract

Breast cancer is one of the most common disease in women. Most of existing breast cancer classification methods include region segmentation, feature extraction and classification phases. It is hard for doctors to understand the conclusion drawn from low level image features. Besides, in cancer hospital more malignant cases than benign cases can be collected, in physical examination center more benign cases can be collected, causing the imbalance problem. To solve above two problems, this study designed a novel breast cancer classification method based on high level Breast Imaging Reporting and Data System (BI-RADS) features. First, an improved Synthetic Minority Oversampling Technique (SMOTE) algorithm is proposed to generate minority samples for balance. Subsequently, coclustering is adopted to mine diagnostic rules. Finally, with Adaboost, the rules can construct a strong classifier. Comparison experiment results on two public datasets shows that the accuracy, precision, recall F1 of proposed method improves more than 5% than comparison methods. Besides, under different imbalance ratios, accuracy of the proposed method is more than 5% higher than comparison methods.

## 1 Introduction

Breast cancer is one of the most common cancers in adult women and more than 30000 deaths are reported in United States every year [1–3]. Nowadays there has been no effective ways to cure it. Fortunately, researchers found that early diagnosis and treatment of breast cancer can offer a high probability of surviving [4–6].

Computer-aided diagnostic (CAD) techniques automatically extract many features from medical images and give the doctors a diagnostic suggestion [7–10]. Many breast ultrasound CAD systems based on artificial intelligence have been proposed in the literature. In recent years, deep learning has attracted much attention because of its high accuracy in image classification. However, deep learning needs very large amounts of training samples. It is difficult to collect enough samples. Related traditional works [10–12] share the same flowchart. There are mainly four steps in the flowchart. The first step is preprocessing, such as denoising, filtering et al. The second step is to segment lesion area from the whole image. The third step is to extract features from lesion area. The last step is to train a classifier to classify the unlabeled samples.

Some of these methods achieved excellent classification accuracy. The most serious limitation is that the features employed by these methods are texture and morphological features. These low level features lack physiological significance. From the perspective of doctors, it is hard to interpret these low level features and the classification results are not convincing.

In practice, doctors usually discriminate benign and malignant lesions with high level descriptive features such as BI-RADS lexicon features [13]. For example, if the echo pattern of the ultrasound breast image is anechoic, it is extremely possible that the breast sample is benign. If the echo pattern is hyperechoic, heterogeneous or hypoechoic, the sample may be malignant. Different doctors may have different experience, therefore discriminating with different diagnosis rules.

Motivated by this phenomenon, we intend to discover diagnosis rule from training samples with coclustering which can cluster both sample dimension and feature dimension simultaneously. In this study, coclustering is first used to mine column nearly constant submatrice [14]. Subsequently apply average strategy to the column of the submatrice, many diagnosis rules can be constructed. After pairwise matching of benign and malignant rules, many weak classifiers can be constructed. Finally, with adaboost, these weak classifiers can be boosted to construct a strong classifier. Besides, the frequently used cocluster quality measure mean square residue score (MSRS) is used to evaluate all kinds of coclusters. Our goal is to find column constant cocluster only. Considering that entropy performs well in measuring similarity of a large set of data, for column nearly constant coclusters, mean entropy score (MES) measure is proposed for cocluster quality assessment.

Moreover, in practice it is rare to collect equal numbers of benign and malignant samples. The predictive capability of classification algorithms is usually impaired by sample imbalance. Nowadays there are mainly two approaches for solving imbalanced data [15,16]. The first is duplicating existing minority samples [17], suffering from overfitting. The second is synthetic minority oversampling technique (SMOTE) [18–20]. SMOTE generates synthetic sample by linearly interpolating a synthetic sample between a randomly selected minority observation and one of its neighboring minority observations. However, randomly linear interpolating is not suitable for processing features having discrete values (BI-RADS feature value is discrete). We proposed an improved SMOTE for generating synthetic samples. Instead of clustering only in the minority samples, the whole samples are automatically clustered with AP cluster [21] to avoid overfitting. Besides, each feature value of the generated minority sample is produced by duplicating the feature value having maximal support among several nearest neighbors instead of randomly linear interpolating.

The main contribution of this study can be summarized as follows:

- BIRADS feature and coclusterring is adopted to mine excellent diagnosis rules.
- A novel oversampling method is proposed to solve imbalance problem.
- Experiment results on two public breast cancer datasets demonstrate the excellent performance of proposed method.

The remaining parts are organized as follows: related works is given in Sect 2, Sect 3 describes preliminary, each step of the proposed method is described in Sect 4, experiment is given in Sect 5.

## 2 Related works

### 2.1 Breast cancer classification

Breast cancer classification is a critical area of research in medical diagnostics, with significant advancements driven by the integration of machine learning and deep learning techniques [22,23]. This section reviews recent studies and methodologies that have contributed to the field.

#### 2.1.1 Traditional methods and early approaches.

Early efforts in breast cancer classification primarily relied on histopathological image analysis and gene expression data. For instance, traditional machine learning methods such as feature selection and pathway-informed classification systems have been explored to improve diagnostic accuracy.

#### 2.1.2 Deep learning approaches.

With the advent of deep learning, convolutional neural networks (CNNs) have become a cornerstone in breast cancer classification. Spanhol et al. demonstrated the effectiveness of CNNs for histopathological image classification, achieving high accuracy rates [24]. More recently, transfer learning has emerged as a powerful technique to leverage pre-trained models for improved classification performance. For example, several studies have utilized transfer learning with models like Inception-ResNet v2 to classify breast cancer subtypes from histopathological images.

#### 2.1.3 Multimodal approaches.

Multimodal deep learning has gained attention by integrating histopathological images with gene expression data. Recent studies have shown that combining these modalities can enhance classification accuracy and provide a more comprehensive understanding of breast cancer subtypes. For instance, a study proposed a weakly supervised lesion detection and diagnosis method using partially annotated ultrasound images, demonstrating the potential of multimodal approaches.

#### 2.1.4 Adversarial networks and data augmentation.

Generative adversarial networks (GANs) have been employed to address challenges such as data imbalance and limited sample sizes. Guan and Loew used GANs in conjunction with transfer learning to improve breast cancer detection using CNNs [25]. Another study applied Wasserstein GAN-based data augmentation to enhance the robustness of classification models.

#### 2.1.5 Transfer learning and ensemble methods.

Transfer learning remains a dominant approach in breast cancer classification due to its ability to adapt pre-trained models to specific tasks. Recent studies have explored novel transfer learning architectures and ensemble methods to further improve classification accuracy. For example, Nair and Subaji proposed a multipath transfer learning approach combined with an ensemble of classifiers to automate breast cancer type identification [26].

### 2.2 Imbalanced classification

Imbalanced data classification is a significant challenge in machine learning, as it often leads to biased models that favor the majority class, resulting in poor performance on minority classes [16,27–29]. This issue is prevalent in various real-world applications, such as fraud detection, medical diagnosis, and fault diagnosis. Over the years, researchers have proposed numerous methods to address this problem, which can be broadly categorized into data-level methods, algorithm-level methods, and hybrid approaches.

#### 2.2.1 Data-level methods.

Data-level methods focus on modifying the training dataset to balance the class distribution. The most common techniques include oversampling, undersampling, and hybrid sampling. Oversampling methods, such as Synthetic Minority Over-sampling Technique (SMOTE), generate synthetic samples for the minority class to increase its representation. SMOTE has been widely adopted and extended in various forms, such as Poly-SMOTE and ProWSyn. Undersampling techniques, on the other hand, reduce the number of samples in the majority class to balance the dataset. However, both oversampling and undersampling have their drawbacks. Oversampling can introduce redundant data and overfitting, while undersampling may lead to loss of important information. Hybrid methods combine both oversampling and undersampling to mitigate these issues [30].

#### 2.2.2 Algorithm-level methods.

Algorithm-level methods aim to improve the performance of classifiers by modifying the learning algorithm itself. One approach is to use cost-sensitive learning, where different misclassification costs are assigned to different classes. Another popular method is to adapt the loss functions to be more sensitive to class imbalance. For example, class-balanced loss functions have been shown to be effective in improving the performance of Gradient Boosting Decision Trees (GBDT) on imbalanced datasets. Additionally, ensemble methods, such as bagging and boosting, have been adapted to handle imbalanced data by dynamically selecting classifiers or using ensemble learning with oversampling [31].

#### 2.2.3 Hybrid methods.

Hybrid methods combine data-level and algorithm-level techniques to leverage the strengths of both approaches. For instance, combining oversampling methods like SMOTE with ensemble learning algorithms has been shown to be effective in improving classification performance. Another example is the use of deep generative models to generate synthetic data for the minority class, followed by training classifiers on the augmented dataset. This approach has demonstrated improved performance in both data synthesis and classification accuracy [32].

## 3 Preliminary

### 3.1 Coclustering

Coclustering(also called biclustering or block-clustering) can cluster data matrix from row dimension and column dimension to find local coherent patterns. Traditional clustering can only cluster from either row dimension or column dimension [33]. Coclustering is firstly proposed to mine local pattern from gene expression data [34]. It has also been widely in many other fields such as stocl analysis [35–37].

**Fig 1:**
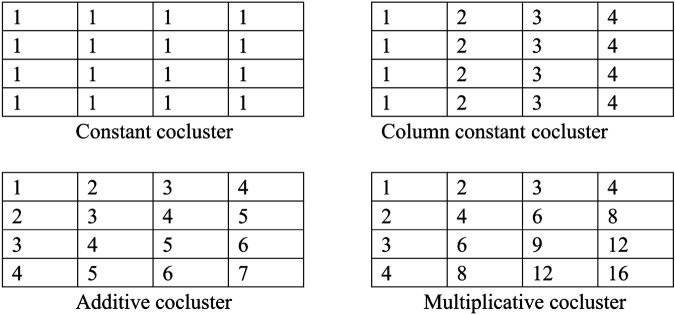
Illustration of coclusters types.

As shown in Fig 1, cocluster can mainly be divided into four types, namely constant cocluster, column constant cocluster, additive cocluster and multiplicative cocluster [38]. In constant cocluster, the value of each element is the same. In column constant cocluster, the value of each element in the same column is the same. In additive cocluster, the difference of each element in each column pair is the same. In multiplicative cocluster,the difference of each element in each column pair is the same.

## 4 Materials and methods

In this study, two public breast ultrasound image datasets are used for evaluation. First dataset is S [39], second dataseet is BUSI [40]. Both datasets contain benign and malignant tumors. Number of benign samples and malignant samples in each dataset are displayed Table 1. Average image size of S is 760×750, average image size of BUSI is 500 × 500. The two public datasets were accessed for research purposes on Jan 1st 2025, authors didn’t have access to information that could identify individual participants during or after data collection.

**Table 1:** Information about dataset.

Dataset	$N_{b}$	$N_{m}$	*N*
S [39]	110	53	163
BUSI [40]	487	210	697

### 4.1 Sample feature description

As shown in Table 2, with doctors’ suggestion, 14 most relative BIRADS features are selected. Each feature has 2 to 6 values. The integer scores in the bracket of the second column are recorded. With doctors’ careful checking, each ultrasound breast tumor image is scored with a vector including 15 elements, left 14 of which are discrete sample feature values and the rightmost one is the sample label.

**Table 2:** Feature descriptors and scoring scheme.

Feature name	Feature value (corresponding discrete scores)
Shape	Oval, Round, Irregular (0,1,2)
Orientation	Parallel, Not parallel (0,1)
Margin integrality	Circumscribed, Not circumscribed (0,1)
Margin ambiguity	Distinct, Indistinct (0,1)
Angular	Absent, Present (0,1)
Microlobulated	Absent, Present (0,1)
Spiculated	Absent, Present (0,1)
Echo pattern	Anechoic, Hyperechoic, Isoechoic, Heterogeneous, Complex cystic and solid, Hypoechoic (0,1,2,3,4,5)
Posterior feature	Enhancement, None, Combined pattern, Shadowing (0,1,2,3)
Calcification in mass	Absent, Coarse, Scattered, Clustered (0,1,2,3)
Architectural distortion	Absent, Present (0,1)
Ducts changes	Normal, Cystic extension, Object found in ducts (0,1,2)
Skin thickening	Absent, Present (0,1)
Edema	Absent, Present (0,1)

### 4.2 Improved SMOTE

The proposed synthetic sample generating method consists of three steps: clustering, filtering, oversampling. First, the entire input space is clustered into optimal number of cluster sets *C* with apcluster [21] which can automatically cluster without predetermining the number of clusters. Subsequently, to avoid oversampling from unsafe areas, only sub clusters *C*_*s*_ where the number of minority samples is large than that of majority samples are selected, ignoring other clusters. Finally, oversampling is performed in each cluster of *C*_*s*_. The minority sample generating ratio *r* is determined with Eq 1. Each cluster *c* in *C*_*s*_ generates n*r synthetic minority samples, where *n* is the number of minority samples in *c*. The oversampling is performed with following steps: (1) randomly select n*r minority samples from cluster *c*. (2) For each selected minority sample *s*, calculate the 5 nearest samples [*s*_1_, *s*_2_,... *s*_5_]. (3 ) Compute each feature value *f*_*i*_ in synthetic sample by copying the value having maximal supports (also called appearing most frequently) among $\left[s_{1}(i),s_{2}(i),...,s_{5}(i)\right]$. *f*_*i*_ is the value of the *i*th feature in the synthetic sample, *i* ranges from 1 to 14.

$$r=\frac{N_{maj}-N_{min}}{N_{csmin}}$$ (1)

where *N*_*maj*_ and *N*_*min*_ are the total number of majority and minority samples in cluster sets *C*, *N*_*csmin*_ is the total number of minority samples in *C*_*s*_.

### 4.3 Coclustering and rule construction

**Fig 2:**
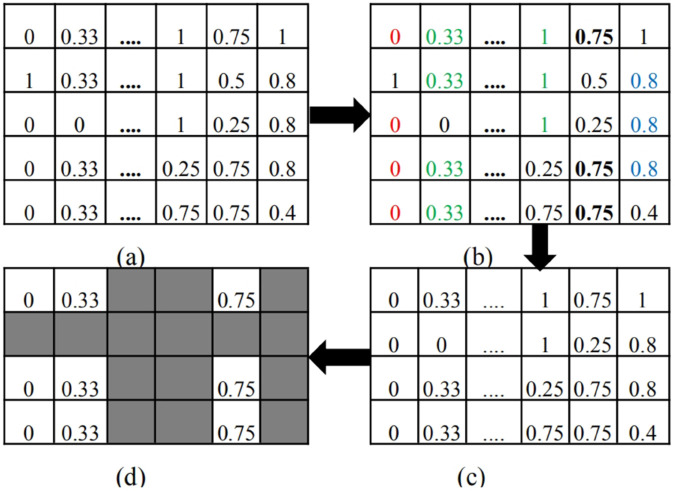
Illustration of searching coclusters (a) Normalized feature matrix (b) cocluster seeds (the elements marked the same color) (c) expand the first cocluster seed (four ‘0’ elements marked red in the first column) to form submatrix (d) final 3*3 cocluster (white submatrix) after iteratively greedily deleting rows and columns having high entropy.

Traditional clustering approaches such as k-means or hierarchical clustering group similar objects together by detecting global similarity. Clustering based on global features contains limited information. Subset of features, namely local information, contain more information. coclustering [41–44] performs well in extracting local information. There are mainly six types of coclusters. Here, we are just interested in constant column cocluster which can transform matrix to horizontal vector. To describe cocluster searching process more graphically, an example of the coclustering process in this context is displayed in Fig 2. The detailed description of each step of the coclustering process is as follows:

1. Due to the values of the same feature in different samples may be in different scale, min-max normalization is applied to each feature column of data matrix 𝐌 to map all the feature values between [0, 1]. 𝐌 is a 586*19 matrix composed of 586 training samples.
2. Apply agglomerative hierarchical clustering (AHC) [45] to each of the left 14 columns of 𝐌 to find similar elements to construct cocluster seeds. The maximum intra-cluster (Mic) great effect in determing the quality of diagnosis rule, its value is determined with grid search.
3. Expand each cocluster seed to the whole columns to form submatrix $M_{s}$. Then iteratively delete the column or row reducing $score(M_{s})$ (Eq 3) most quickly until the stopping criteria is met. The stopping criteria is that $score(M_{s})$ is less than predetermined threshold *delta* which is the maximum allowable dissimilarity. The value of *delta* is determined with grid search. To obtain robust diagnosis rules, the size of the cocluster cannot be too small. All the coclusters containing less than 5 rows or less than 3 columns are deleted.
  $$e_{j}=-\sum_{i=1}^{k}\frac{N(i)}{r}log\frac{N(i)}{r}$$ (2)
  where *e*_*j*_ is the entropy of column *j*, *k* is the number of clusters in one column, *N*(*i*) is the number of elements in the *i*th cluster, *r* is the number of the rows in $M_{s}$.
  $$score(M_{s})=\frac{\sum_{j=1}^{c}e_{j}}{c}$$ (3)
  where $score(M_{s})$ is entropy score of $M_{s}$, *c* is the number of columns in matrix $M_{s}$, *e*_*j*_ is calculated with Eq 2.

Having obtained many coclusters from above step, column average and majority voting strategy is adopted to transform the coclusters to diagnosis rule vectors. Average the column of the cocluster to obtain the preconditions of rule. To determine the postcondition (benign or malignant), the majority voting strategy is adopted. Assume *N* is the number of samples in $M_{s}$, *N*_*b*_, *N*_*m*_ denote the number of samples corresponding to benign and malignant label respectively. If $max(N_{b},N_{m})$ is greater than 0.65*N, corresponding label having maximum supports is assigned as the label of the rule. Otherwise, it is not a good cocluster and discard it. To obtain a robust rule, the winning rate is set as 0.65 instead of 0.5 which is a common threshold in voting problem in the literature.

### 4.4 Weak classifiers construction

**Fig 3:**
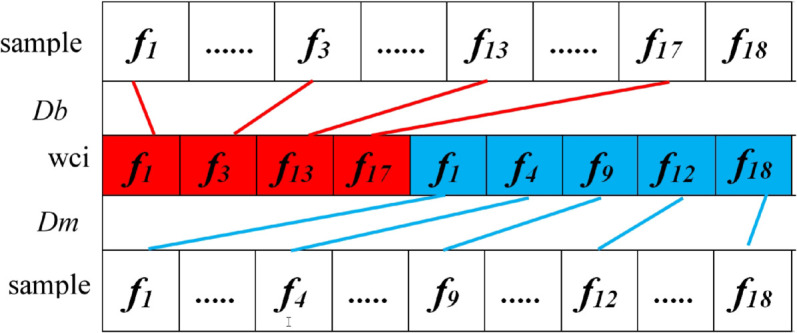
Illustration of the working principle of weak classifier.

Assuming *l* benign rules and *k* malignant rules have been obtained from above steps. In this study, one benign rule by one malignant rule pair strategy is utilized to build weak classifier. Totally l*k weak classifiers can be obtained. As shown in Fig 3, when a test sample is input to weak classifier wci, the distance between the test sample and benign rule (marked red), malignant rule (marked blue) of wci is computed as Eq 4, Eq 5 respectively. If *D*_*b*_ is smaller than *D*_*m*_, then test sample is predicted as benign, and malignant otherwise.

$$D_{b}=\frac{{\left\|f_{tb}-f_{b}\right\|}_{2}}{{\left\|f_{maxb}-f_{minb}\right\|}_{2}}$$ (4)

$$D_{m}=\frac{{\left\|f_{tm}-f_{m}\right\|}_{2}}{{\left\|f_{maxm}-f_{minm}\right\|}_{2}}$$ (5)

In Eq 4, *f*_*b*_ is a horizontal vector containing the whole features of benign rule in wci, *f*_*tb*_ is a vector containing the features in test sample having the same indexes as that in *f*_*b*_. *f*_*maxb*_ and *f*_*minb*_ are two vectors containing the maximum and minimum of the columns in *M* having the same indexes as that in *f*_*b*_. The variables in Eq 5 share similar definitions as Eq 4. *f*_*b*_ and *f*_*m*_ are different subsets of the whole 18 features. The distance calculation in different feature subspaces is a challenging task.

### 4.5 Strong classifier construction

In this phase many weak classifiers have been constructed, adaboost is utilized to build strong classifier. The adaboost method in this study is the same as that in [46] except for the number of iterations is set as the number of weak classifiers.

The final strong classifier can be expressed as:

$$PL(s)=sign(\sum_{t=1}^{N}w_{t}*wc_{t}(s))$$ (6)

where *PL*(*s*) is the predicted label of test sample *s*, *w*_*t*_ is the weight of the *t*th weak classifier *wc*_*t*_, *wc*_*t*_(*s*) is the output of *wc*_*t*_ with test sample *s* as input.

## 5 Results

### 5.1 Experiment setup

To evaluate the performance of proposed methods, comparison experiments were conducted. Since the proposed method contains Improved SMOTE, coclustering and ADaboost, it is named as ISCCAD. The first comparison algorithm is baseline algorithm SVM [47]. The second comparison algorithm is NPC (Neighbors Progressive Competition) [48]. NPC is an excellent algorithm for solving the class imbalance problem [48]. In NPC, every training sample is given a grade value. Then, for classifying the test sample: sum grades in each class until one class’s accumulated value is marginally bigger than the others’, finally the classifier assigns the winner class to the test sample. NPC considers progressively more nearest neighbors as long as the two classes are close in grades. The remaining three comparison algorithm are VGG16 [49], LeNet [50] and ResNet [51], the three algorithms are frequently used CNN based methods.

All methods are implemented with Matlab language, Experiments are run on a laptop with i5-8350U CPU, windows 11 operating system, 16GB memory, 512GB disk.

In this study, two public breast ultrasound image datasets in Table 1 are used for evaluation. Randomly select 80% samples for training and remaining 20% samples for testing. To ensure imbalance, the ratio of majority samples in training datasets are kept over 60%. Results are obtained by 10-fold cross-validation. All the experiments are implemented in matlab programming language. As shown in Eqs 7–10, four frequently used metrics, namely accuracy, precision, recall and F1 [52] are used for performance evaluation.

$$Accuracy=\frac{TP+TN}{TP+TN+FP+FN}$$ (7)

$$Precision=\frac{TP}{TP+FP}$$ (8)

$$Recall=\frac{TP}{TP+FN}$$ (9)

$$F1=2\times \frac{\text{Precision}\times \text{Recall}}{\text{Precision}+\text{Recall}}$$ (10)

where *TP* is the number of correctly recognized positive samples, *TN* is the number of correctly recognized negative samples, *FP* is the number of negative samples that is wrongly recognized positive samples, *FN* is the number of positive samples that is wrongly recognized negative samples.

### 5.2 Experiment results

Four experiments are conducted in this study to explore the performance of ISCCAD.

#### 5.2.1 Overall result.

**Fig 4:**
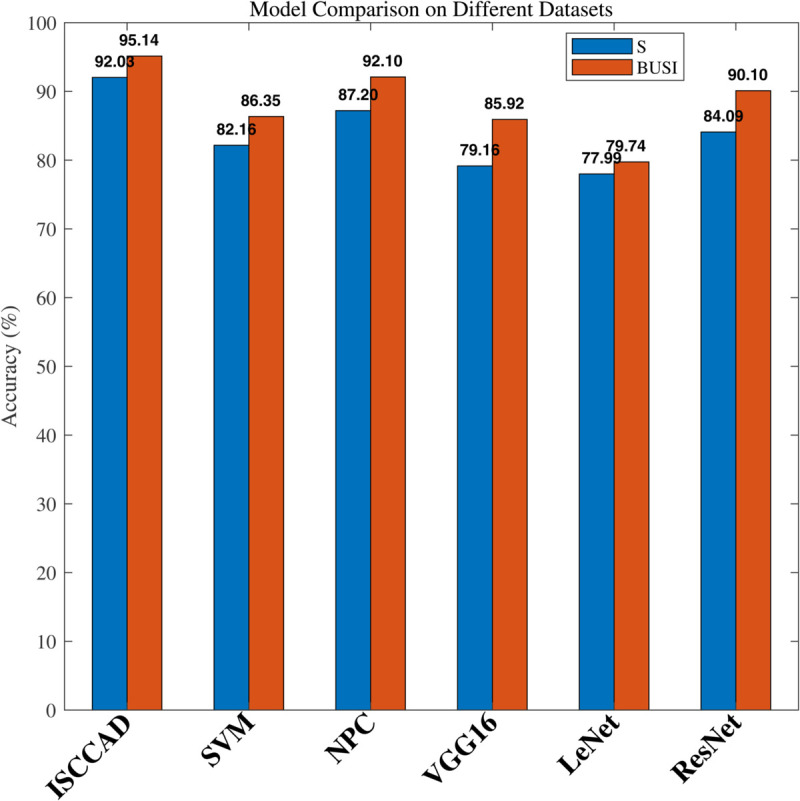
Accuracy of six methods on two datasets.

**Fig 5:**
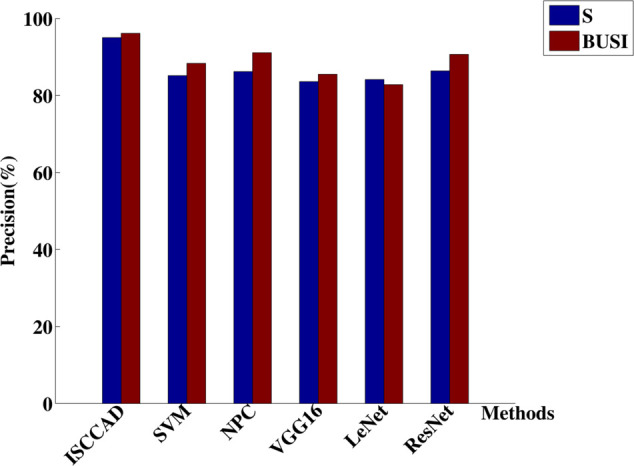
Precision of six methods on two datasets.

**Fig 6:**
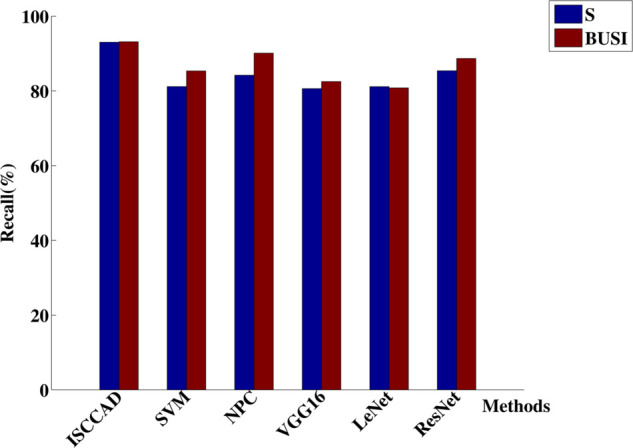
Recall of six methods on two datasets.

**Fig 7:**
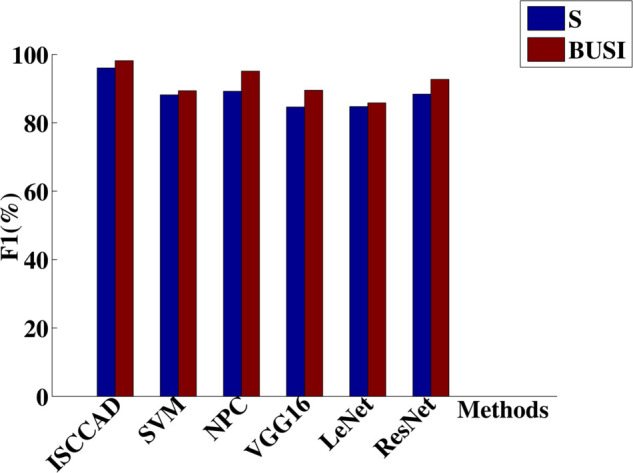
F1 of six methods on two datasets.

Experiment results are summarized in Figs 4–7. The four metrics from 80/20 hold-out repeated 10 times runnings of the algorithm with different random partitions of train and test samples did not differ statistically. It can be seen that ISCCAD achieved better results than other five methods, demonstrating the superiority of ISCCAD. The generated diagnostic rules are validated by doctors, they think the rules are good and are consistent with their clinical experience. Paired t-test is adopted for significance test, under 0.95 confidence level [53], as shown in Table 3, confidence interval(CI) of ISCCAD outperforms other methods.

**Table 3:** Accuracy confidence interval comparison on dataset S.

Method	ISCCAD	SVM	NPC	VGG16	LeNet	ResNet
CI(%)	[92.6, 92.8]	[81.3, 81.9]	[86.1, 87.3]	[79.1, 79.6]	[77.2, 73.4]	[85.1, 86.3]]

Analyzing the whole found benign rules, many frequent phenomena can be found. For example, *f*_14_ (0) (corresponding physiological meaning is Edema(Absent)) appear simultaneously in the whole benign rules, *f*_1_ (0) (corresponding physiological meaning is Shape (Oval)) appears in the whole malignant rules. We analyze the found biclusters, finding that oval shape and parallel lesion orientation usually appear simultaneously in benign samples; parallel lesion orientation and circumscribed and distinct border usually appear simultaneously in benign samples. Irregular shape and non-parallel orientation usually appear simultaneously in malignant samples. These phenomena are in accordance with doctors’ diagnosis experience.

It is observed that the more rows in the cocluster, the higher the weight of corresponding weak classifier in the strong classifier. Average MES of the coclusters mined from the imbalanced and balanced data is 0.0082 and 0.0075, respectively. Compared with original imbalanced dataset, more coclusters are mined from the balanced dataset containing synthetic samples.

#### 5.2.2 Effect of imbalance ratios.

To comprehensively explore the performance of ISCCAD under different imbalance ratios(IR), imbalance performance experiment is conducted. When varying imbalance ratio, the test set remained unchanged, only train set are randomly sampled. IR is calculated with Eq 11. In this study, as shown in Table 4, ten imbalance ratios are set by selecting subset from majority class.

$$IR=\frac{N_{max}-N_{min}}{N_{min}}$$ (11)

where *N*_*max*_ is the sample number of majority class, *N*_*min*_ is the number of minority class.

**Table 4:** Imbalance ratio seperation.

No	$N_{b}$	$N_{m}$	IR
1	477	210	1.27
2	459	210	1.18
3	432	210	1.06
4	404	210	0.92
5	376	210	0.79
6	349	210	0.66
7	321	210	0.53
8	293	210	0.39
9	265	210	0.26
10	238	210	0.13

**Fig 8:**
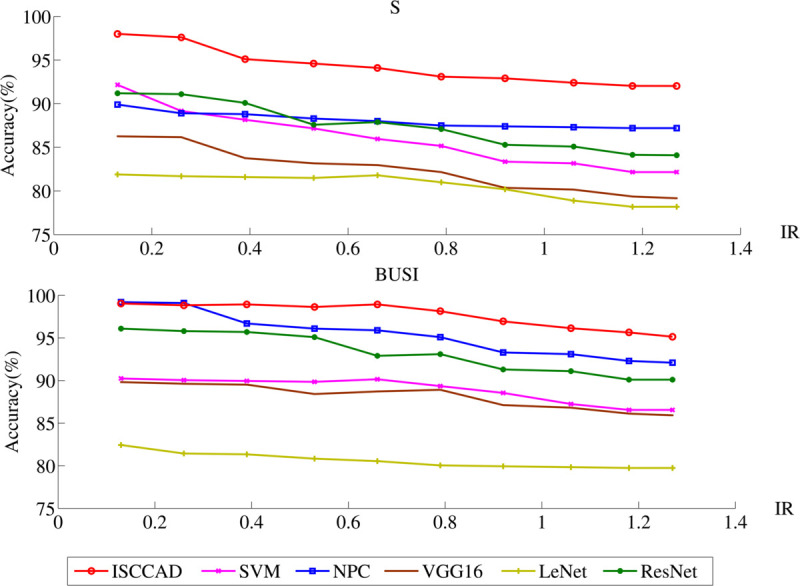
Accuracy of six methods on two datasets under different IR.

The classification of all methods on two datasets under different all IR is displayed in Fig 8. It can be found that under all IR, the accuracy of ISCCAD is the highest. With the increasing of IR, accuracy of all methods drop.

#### 5.2.3 Runtime analysis.

Besides, training time and testing time is another measure that reflect method’s performance. Training time and testing time comparison of all methods in BUSI dataset is shown in Table 5. It can be found that ISCAAD obtains the shortest training time and testing time.

**Table 5:** Time for training and testing for each method in BUSI.

Methods	Training time	Testing time
ISCAAD	78 ms	6 us
SVM	191 ms	26 us
NPC	381 ms	96 us
VGG16	6.1 s	5.6 ms
LeNet	3 s	4.1 ms
ResNet	5.2 s	5.1 ms

Additional comparison experiments on kaggle breast cancer dataset BCC (https://www.kaggle.com/code/niteshyadav3103/breast-cancer-classification), experiment results on Table 6 demonstrate the superiority of proposed method. Standard oversampling method SO [54] is also added for comparison.

**Table 6:** Accuracy of all methods on BCC.

Methods	ISCCAD	SVM	NPC	VGG16	LeNet	ResNet	SO
Accuracy	96.46%	89.3%	93.1%	84.81%	78.77%	90.11%	80.31%

#### 5.2.4 Ablation study.

Since the the contribution of the study lies in two parts: improved SMOTE for minority sample generation and coclustering adaboost for recognition, ablation study is conducted to verify the effectiveness of each contribution. For improved SMOTE, its effectiveness is demonstrated by comparing ISCAAD with CCAD which is generated by deleting improved SMOTE from ISCAAD. For coclustering adaboost, its effectiveness is demonstrated by comparing ISCAAD with ISSVM which is generated by replacing coclustering adaboost with SVM.

**Fig 9:**
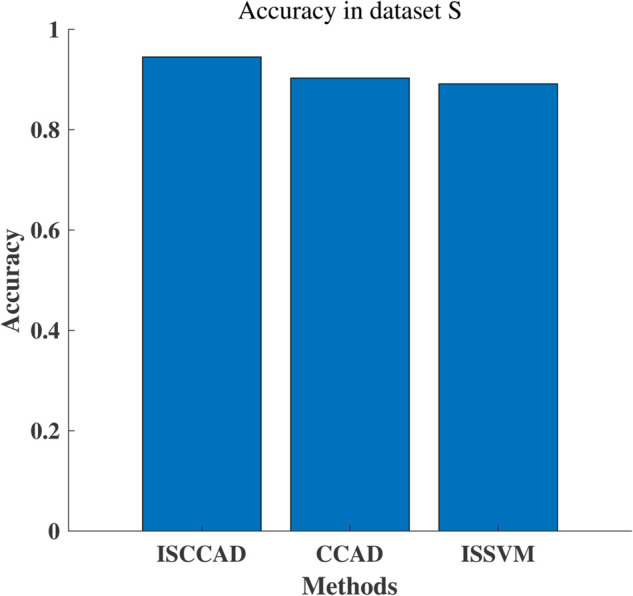
Ablation study result.

The ablation result in BUSI dataset is shown in Fig 9. It can be found that ISCCAD obtains the highest accuracy, the positive contribution of improved SMOTE and coclustering adaboost are verified.

## 6 Conclusion

In this study, a novel imbalanced ultrasound breast cancer image classification method is proposed. An Improved SMOTE is investigated for imbalanced data problem. Considering that the classification results predicted from texture and morphological features lack of interpretability, high level BI-RADS features are utilized. coclustering is employed to mine diagnosis rules that is in accordance with doctors’ practical diagnosis methods. To assess the quality of column nearly constant cocluster, MES measure is proposed. Comparison experiments on two public datasets validated the superiority of proposed method. The limitation is that the feature value of all samples are given by doctors, appropriate automatical feature scoring scheme should be designed to alleviate doctors’ burden. It is anticipated to be a reference method for future studies in imbalanced medical image classification task. In the future, automating feature extraction with deep learning should be done.

## References

[1] Lupat R, Perera R, Loi S, Li J. Moanna: multi-omics autoencoder-based neural network algorithm for predicting breast cancer subtypes. IEEE Access. 2023;11:10912–24. doi: 10.1109/access.2023.3240515

[2] Hamed G, Marey M, Amin SE, Tolba MF. Automated breast cancer detection and classification in full field digital mammograms using two full and cropped detection paths approach. IEEE Access. 2021;9:116898–913. doi: 10.1109/access.2021.3105924

[3] Chen C, Wang Y, Niu J, Liu X, Li Q, Gong X. Domain knowledge powered deep learning for breast cancer diagnosis based on contrast-enhanced ultrasound videos. IEEE Trans Med Imaging. 2021;40(9):2439–51. doi: 10.1109/TMI.2021.3078370 33961552

[4] Elsheakh DN, Fahmy OM, Farouk M, Ezzat K, Eldamak AR. An early breast cancer detection by using wearable flexible sensors and artificial intelligent. IEEE Access. 2024;12:48511–29. doi: 10.1109/access.2024.3380453

[5] Elouerghi A, Bellarbi L, Errachid A, Yaakoubi N. An IoMT-based wearable thermography system for early breast cancer detection. IEEE Trans Instrum Meas. 2024;73:1–17. doi: 10.1109/tim.2024.3435184

[6] Batool A, Byun Y-C. Toward improving breast cancer classification using an adaptive voting ensemble learning algorithm. IEEE Access. 2024;12:12869–82. doi: 10.1109/access.2024.3356602

[7] Fontanellaz M, Christe A, Christodoulidis S, Dack E, Roos J, Drakopoulos D, et al. Computer-aided diagnosis system for lung fibrosis: from the effect of radiomic features and multi-layer-perceptron mixers to pre-clinical evaluation. IEEE Access. 2024;12:25642–56. doi: 10.1109/access.2024.3350430

[8] Xie X, Tian Y, Ota K, Dong M, Liu Z, Jin H, et al. Reinforced computer-aided framework for diagnosing thyroid cancer. IEEE/ACM Trans Comput Biol Bioinform. 2024;21(4):737–47. doi: 10.1109/TCBB.2023.3251323 37028014

[9] Nour MK, Issaoui I, Edris A, Mahmud A, Assiri M, Ibrahim SS. Computer aided cervical cancer diagnosis using gazelle optimization algorithm with deep learning model. IEEE Access. 2024;12:13046–54. doi: 10.1109/access.2024.3351883

[10] Guo J, Yuan H, Shi B, Zheng X, Zhang Z, Li H, et al. A novel breast cancer image classification model based on multiscale texture feature analysis and dynamic learning. Sci Rep. 2024;14(1):7216. doi: 10.1038/s41598-024-57891-5 38538814 PMC10973442

[11] Mostefaoui GK, Islam SR, Tariq F. Artificial intelligence for disease diagnosis and prognosis in smart healthcare. CRC Press; 2023.

[12] Zhu Z, Wang S-H, Zhang Y-D. A survey of convolutional neural network in breast cancer. Comput Model Eng Sci. 2023;136(3):2127–72. doi: 10.32604/cmes.2023.025484 37152661 PMC7614504

[13] Obenauer S, Hermann KP, Grabbe E. Applications and literature review of the BI-RADS classification. Eur Radiol. 2005;15(5):1027–36. doi: 10.1007/s00330-004-2593-9 15856253

[14] Huang N, Xiao L, Liu J, Chanussot J. Graph convolutional sparse subspace coclustering with nonnegative orthogonal factorization for large hyperspectral images. IEEE Trans Geosci Remote Sensing. 2022;60:1–16. doi: 10.1109/tgrs.2021.3096320

[15] Rezvani S, Wang X. A broad review on class imbalance learning techniques. Applied Soft Computing. 2023;143:110415. doi: 10.1016/j.asoc.2023.110415

[16] Ahmed Z, Issac B, Das S. Ok-NB: an enhanced OPTICS and k-naive bayes classifier for imbalance classification with overlapping. IEEE Access. 2024;12:57458–77. doi: 10.1109/access.2024.3391749

[17] Eltayeb RY, Karrar AE, Osman WI, Ali MM. Handling imbalanced data through re-sampling: systematic review. IJEEI. 2023;11(2):503–14. doi: 10.52549/.v11i2.4471

[18] Chaerul Ekty Saputra D, Sunat K, Ratnaningsih T. SMOTE-MRS: a novel SMOTE-multiresolution sampling technique for imbalanced distribution to improve prediction of anemia. IEEE Access. 2024;12:154675–99. doi: 10.1109/access.2024.3482968

[19] Dablain D, Krawczyk B, Chawla NV. DeepSMOTE: fusing deep learning and SMOTE for imbalanced data. IEEE Trans Neural Netw Learn Syst. 2023;34(9):6390–404. doi: 10.1109/TNNLS.2021.3136503 35085094

[20] Azhar NA, Mohd Pozi MS, Mohamed Din A, Jatowt A. An investigation of SMOTE based methods for imbalanced datasets with data complexity analysis. IEEE Trans Knowl Data Eng. 2022;:1–1. doi: 10.1109/tkde.2022.3179381

[21] Hidayatulloh A, Bamufleh S, Chaabani A, Elfeki A, Al-Wagdany A. Clustering similar ungauged hydrologic basins in saudi arabia by message passing algorithms. Earth Syst Environ. 2024;8(2):325–45. doi: 10.1007/s41748-024-00379-z

[22] Navaneethakrishnan R, Alagumeenaakshi M, Ajay V, Shawkat TB, Priya SB. Breast cancer diagnosis through soft computing approaches: a survey. In: 2021 International Conference on Advancements in Electrical, Electronics, Communication, Computing and Automation (ICAECA). 2021. p. 1–4.

[23] Hayum AA, Jaya J, Sivakumar R, Paulchamy B. Critical review on breast cancer classification. In: 2022 IEEE International Conference on Distributed Computing and Electrical Circuits and Electronics (ICDCECE). IEEE; 2022. p. 1–6.

[24] Spanhol FA, Oliveira LS, Petitjean C, Heutte L. Breast cancer histopathological image classification using convolutional neural networks. In: 2016 International Joint Conference on Neural Networks (IJCNN). 2016. p. 2560–7. 10.1109/ijcnn.2016.7727519

[25] Shams S, Platania R, Zhang J, Kim J, Lee K, Park SJ. Deep generative breast cancer screening and diagnosis. In: International conference on medical image computing and computer-assisted intervention. Springer; 2018. p. 859–67.

[26] Chowdhury P, El-Dosuky M, Kamel S. Breast cancer detection using deep learning on biomedical mammogram images. J Theor Appl Inf Technol. 2024;102(7):2924.

[27] Li S, Song L, Wu X, Hu Z, Cheung Y, Yao X. Multi-class imbalance classification based on data distribution and adaptive weights. IEEE Trans Knowl Data Eng. 2024;36(10):5265–79. doi: 10.1109/tkde.2024.3384961

[28] Ochal M, Patacchiola M, Vazquez J, Storkey A, Wang S. Few-shot learning with class imbalance. IEEE Trans Artif Intell. 2023;4(5):1348–58. doi: 10.1109/tai.2023.3298303

[29] Ebenuwa SH, Sharif MS, Alazab M, Al-Nemrat A. Variance ranking attributes selection techniques for binary classification problem in imbalance data. IEEE Access. 2019;7:24649–66. doi: 10.1109/access.2019.2899578

[30] Wang L, Han M, Li X, Zhang N, Cheng H. Review of classification methods on unbalanced data sets. IEEE Access. 2021;9:64606–28. doi: 10.1109/access.2021.3074243

[31] Abokadr S, Azman A, Hamdan H, Amelina N. Handling imbalanced data for improved classification performance: methods and challenges. In: 2023 3rd International Conference on Emerging Smart Technologies and Applications (eSmarTA). 2023. p. 1–8. 10.1109/esmarta59349.2023.10293442

[32] Aryuni M, Fatichah C, Yuniarti A. Resampling methods for imbalanced datasets in multi-label classification: a review. In: 2024 IEEE 14th Symposium on Computer Applications & Industrial Electronics (ISCAIE). 2024. p. 472–7. 10.1109/iscaie61308.2024.10576243

[33] Huang D, Deng X, Chen D-H, Wen Z, Sun W, Wang C-D, et al. Deep clustering with hybrid-grained contrastive and discriminative learning. IEEE Trans Circuits Syst Video Technol. 2024;34(10):9472–83. doi: 10.1109/tcsvt.2024.3399596

[34] Qian S, Liu H, Yuan X, Wei W, Chen S, Yan H. Row and column structure-based biclustering for gene expression data. IEEE/ACM Trans Comput Biol Bioinform. 2022;19(2):1117–29. doi: 10.1109/TCBB.2020.3022085 32894722

[35] Huang Q, Yang J, Feng X, Liew AW-C, Li X. Automated trading point forecasting based on bicluster mining and fuzzy inference. IEEE Trans Fuzzy Syst. 2020;28(2):259–72. doi: 10.1109/tfuzz.2019.2904920

[36] Cotelo JM, Ortega FJ, Troyano JA, Enriquez F, Cruz FL. Known by who we follow: a biclustering application to community detection. IEEE Access. 2020;8:192218–28. doi: 10.1109/access.2020.3032015

[37] Neves F, Finamore AC, Madeira SC, Henriques R. Mining actionable patterns of road mobility from heterogeneous traffic data using biclustering. IEEE Trans Intell Transport Syst. 2022;23(7):6430–45. doi: 10.1109/tits.2021.3057240

[38] Sun J, Zhang Y. Recommendation system with biclustering. Big Data Min Anal. 2022;5(4):282–93. doi: 10.26599/bdma.2022.9020012

[39] Yap MH, Pons G, Marti J, Ganau S, Sentis M, Zwiggelaar R, et al. Automated breast ultrasound lesions detection using convolutional neural networks. IEEE J Biomed Health Inform. 2018;22(4):1218–26. doi: 10.1109/JBHI.2017.2731873 28796627

[40] Al-Dhabyani W, Gomaa M, Khaled H, Fahmy A. Dataset of breast ultrasound images. Data Brief. 2019;28:104863. doi: 10.1016/j.dib.2019.104863 31867417 PMC6906728

[41] Huang N, Xiao L, Xu Y, Chanussot J. A bipartite graph partition-based coclustering approach with graph nonnegative matrix factorization for large hyperspectral images. IEEE Trans Geosci Remote Sensing. 2022;60:1–18. doi: 10.1109/tgrs.2021.3097358

[42] Banerjee B, Buddhiraju KM. Domain adaptation in the absence of source domain labeled samples—a coclustering-based approach. IEEE Geosci Remote Sensing Lett. 2016;13(12):1905–9. doi: 10.1109/lgrs.2016.2617199

[43] Pizzuti C, Rombo SE. A coclustering approach for mining large protein-protein interaction networks. IEEE/ACM Trans Comput Biol Bioinform. 2012;9(3):717–30. doi: 10.1109/TCBB.2011.158 22201069

[44] Lijun Zhang, Chun Chen, Jiajun Bu, Zhengguang Chen, Deng Cai, Jiawei Han. Locally discriminative coclustering. IEEE Trans Knowl Data Eng. 2012;24(6):1025–35. doi: 10.1109/tkde.2011.71

[45] Ran X, Xi Y, Lu Y, Wang X, Lu Z. Comprehensive survey on hierarchical clustering algorithms and the recent developments. Artif Intell Rev. 2022;56(8):8219–64. doi: 10.1007/s10462-022-10366-3

[46] Xu M, Baraldi P, Lu X, Zio E. Generative adversarial networks with AdaBoost ensemble learning for anomaly detection in high-speed train automatic doors. IEEE Trans Intell Transport Syst. 2022;23(12):23408–21. doi: 10.1109/tits.2022.3203871

[47] Yan Y, Le X, Yang T, Yu H. Interpretable PCA and SVM-based leak detection algorithm for identifying water leakage using SAR-derived moisture content and InSAR closure phase. IEEE J Sel Top Appl Earth Observations Remote Sensing. 2024;17:15136–47. doi: 10.1109/jstars.2024.3443127

[48] Saryazdi S, Nikpour B, Nezamabadi-Pour H. NPC: Neighbors’ progressive competition algorithm for classification of imbalanced data sets. In: 2017 3rd Iranian Conference on Intelligent Systems and Signal Processing (ICSPIS). 2017. p. 28–33. 10.1109/icspis.2017.8311584

[49] Albashish D, Al-Sayyed R, Abdullah A, Ryalat MH, Ahmad Almansour N. Deep CNN model based on VGG16 for breast cancer classification. In: 2021 International Conference on Information Technology (ICIT). 2021. p. 805–10. 10.1109/icit52682.2021.9491631

[50] Lecun Y, Bottou L, Bengio Y, Haffner P. Gradient-based learning applied to document recognition. Proc IEEE. 1998;86(11):2278–324. doi: 10.1109/5.726791

[51] He K, Zhang X, Ren S, Sun J. Deep residual learning for image recognition. In: 2016 IEEE Conference on Computer Vision and Pattern Recognition (CVPR). 2016. p. 770–8. 10.1109/cvpr.2016.90

[52] Al-Badri AH, Ismail NA, Al-Dulaimi K, Rehman A, Abunadi I, Bahaj SA. Hybrid CNN model for classification of rumex obtusifolius in grassland. IEEE Access. 2022;10:90940–57. doi: 10.1109/access.2022.3200603

[53] Petiau RB. Confidence interval estimation for short-term load forecasting. In: 2009 IEEE Bucharest PowerTech. 2009. p. 1–6.

[54] Henning P, Peterseim D. Oversampling for the multiscale finite element method. Multiscale Model Simul. 2013;11(4):1149–75. doi: 10.1137/120900332

